# Reoperation for a giant arch anastomotic pseudoaneurysm eleven years after total arch replacement with island reconstruction

**DOI:** 10.1186/s13019-018-0694-9

**Published:** 2018-01-15

**Authors:** Ryohei Matsuura, Yasushi Tsutsumi, Osamu Monta, Hisazumi Uenaka, Kenji Tanaka, Takaaki Samura, Hirokazu Ohashi

**Affiliations:** 1 Department of Cardiovascular Surgery, Fukui Cardiovascular Center, 2-228 Shinbo, Fukui, 910-0833 Japan; 2 Department of Cardiovascular Surgery, Osaka University Graduate School of Medicine, 2-2 E1, Yamadaoka, Suita-shi, Osaka 565-0871 Japan

**Keywords:** Island reconstruction, Pseudoaneurysm, Arch replacement

## Abstract

**Background:**

The long-term effects of some surgical treatment procedures of arch replacement for aortic dissection or aortic aneurysm are unknown.

**Case presentation:**

The present study reports the case of a 68-year-old man admitted to our hospital for aortic arch anastomotic pseudoaneurysm with concomitant aortic root enlargement and coronary artery stenosis. Eleven years ago, at the age of 56 years, he underwent total arch replacement with island reconstruction for chronic aortic dissection. We performed a second total arch replacement, aortic root replacement, and coronary artery bypass, using a cardiopulmonary bypass with cannulation through the right subclavian artery, femoral artery, and femoral vein prior to re-sternotomy. We also used selective cerebral perfusion. Postoperatively, the patient temporarily required reintubation; however, he was discharged in good condition on the fiftieth postoperative day.

**Conclusions:**

This case suggests that island reconstruction has the potential to cause arch anastomotic pseudoaneurysms, particularly after a long postoperative period.

## Background

Along with recent progress in aortic surgery, the number of patients undergoing thoracic aortic aneurysm surgery has increased, with evident improvements in long-term performance [1, 2]. However, some cases require reoperation long after the initial surgery [3–5]. We experienced a rare case of pseudoaneurysm in the arch anastomotic region in the eleventh year after a total arch replacement with island reconstruction.

## Case presentation

**Fig. 1:**
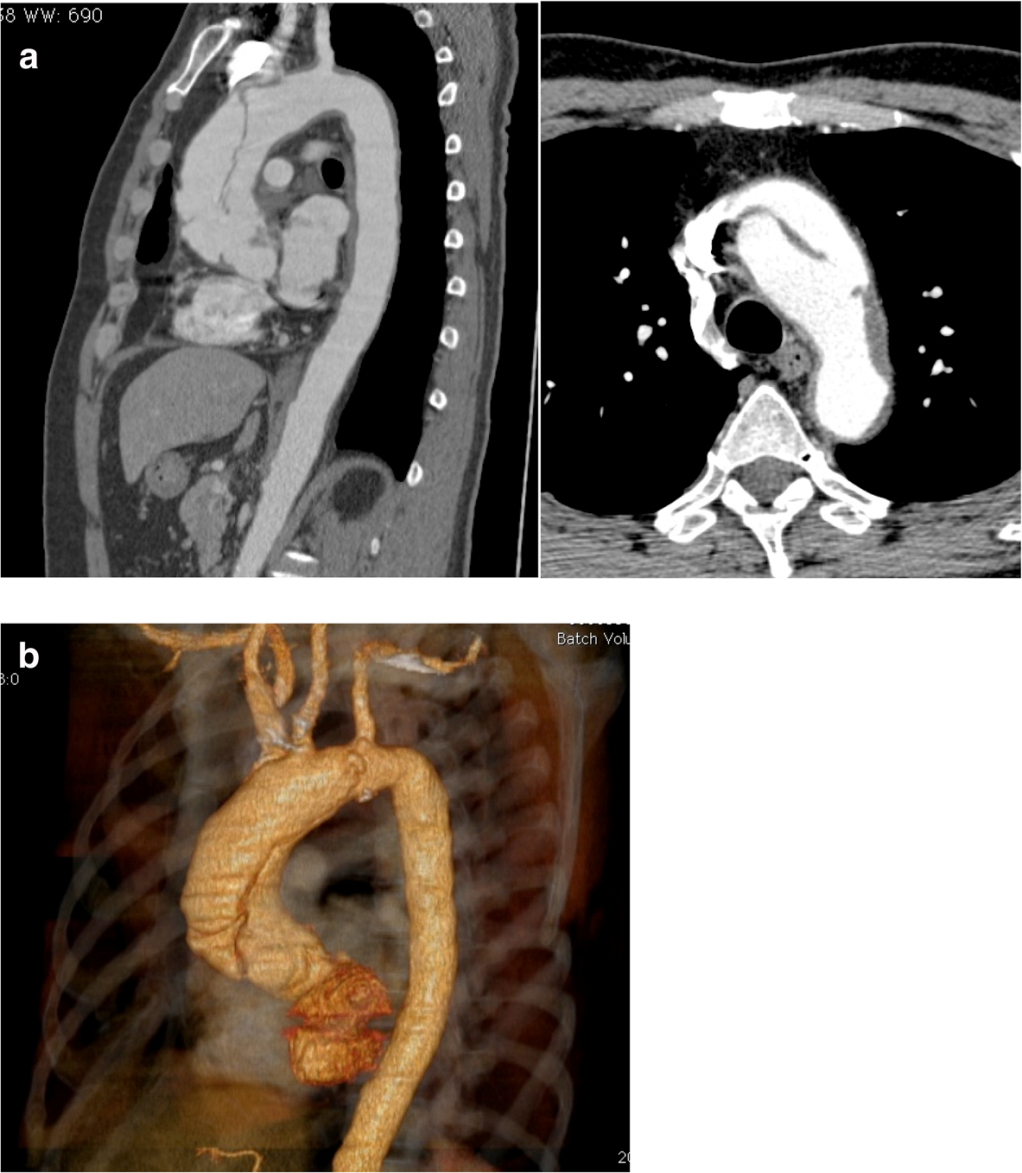
CT scan showing a Stanford type A aortic dissection. **a** Multiplanar reconstruction view of CT angiogram of the thoracic aorta. The maximum diameter of the ascending aorta was 51 mm. The entry was at the ascending aorta and the reentry was between the left common carotid artery and left subclavian artery. **b** Preoperative multi detector computed tomography

**Fig. 2:**
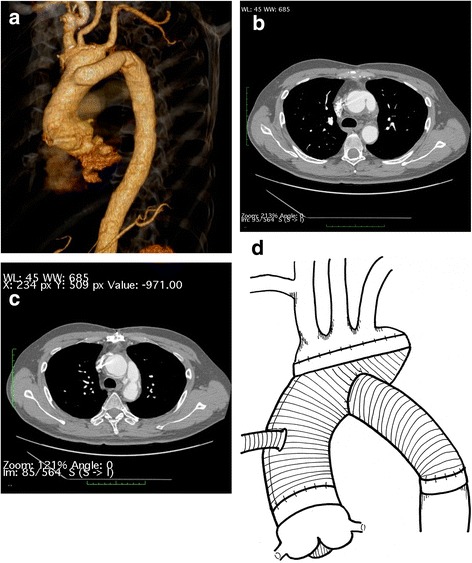
Postoperative multidetector computed tomography (MDCT) showing total aortic arch replacement with island reconstruction using the T-graft technique. It also shows that no false lumens remained after the first surgery. **a** Synthetic image, (**b**-**c**) axial view, and (**d**) scheme of the procedure. The diameter of the graft: proximal, 30 mm; distal, 18 mm

A 68-year-old man was admitted to our hospital due to aortic arch anastomotic pseudoaneurysm with concomitant aortic root enlargement and coronary artery stenosis. At 56 years of age, the patient had undergone a total arch replacement at another hospital, with three-branched island reconstruction performed using the T-graft technique for a chronic aortic dissection, expanding from the ascending aorta to distal aortic arch **(**Figs. 1 and 2**)**. In the aortic arch island reconstruction, the adventitia and intima of the arteries were combined and reinforced externally by a felt, and end-to-end anastomosis was performed with a 30-mm synthetic vascular graft with one branch. Thereafter, the patient was followed up by a local physician; however, a computed tomography (CT) scan was not performed, as no relevant symptoms were observed. A CT scan at another hospital, however, showed a giant pseudoaneurysm at the site of the island reconstruction 11 years later. Though endovascular repair was considered, the patient was ultimately determined to be inoperable by the other hospital, as enlargement of the aortic root was also observed. He was subsequently referred to our hospital and he opted to undergo surgery at our institution.

At the time of presentation, the patient was 168 cm in height and weighed 71 kg. His vital signs were as follows: body temperature, 36.1 °C; blood pressure, 120/60 mmHg (no difference between left and right arms); pulse rate, 70 bpm (regular); and SpO_2_, 100% (room air). Upon physical examination, his consciousness was clear, but expressed hoarseness in his voice. The patient had a median sternotomy scar. A diastolic murmur was detected in the third intercostal space at the left sternal border on thoracic auscultation. Breath sounds were normal, and the abdomen was soft and flat. An electrocardiogram showed normal sinus rhythm. A chest X-ray revealed enlargement of the left 1st arch and elevation of the left diaphragm. His cardio-thoracic ratio was 55.4%.

There were no abnormal laboratory findings nor did echocardiography reveal abnormal cardiac function upon admission.

**Fig. 3:**
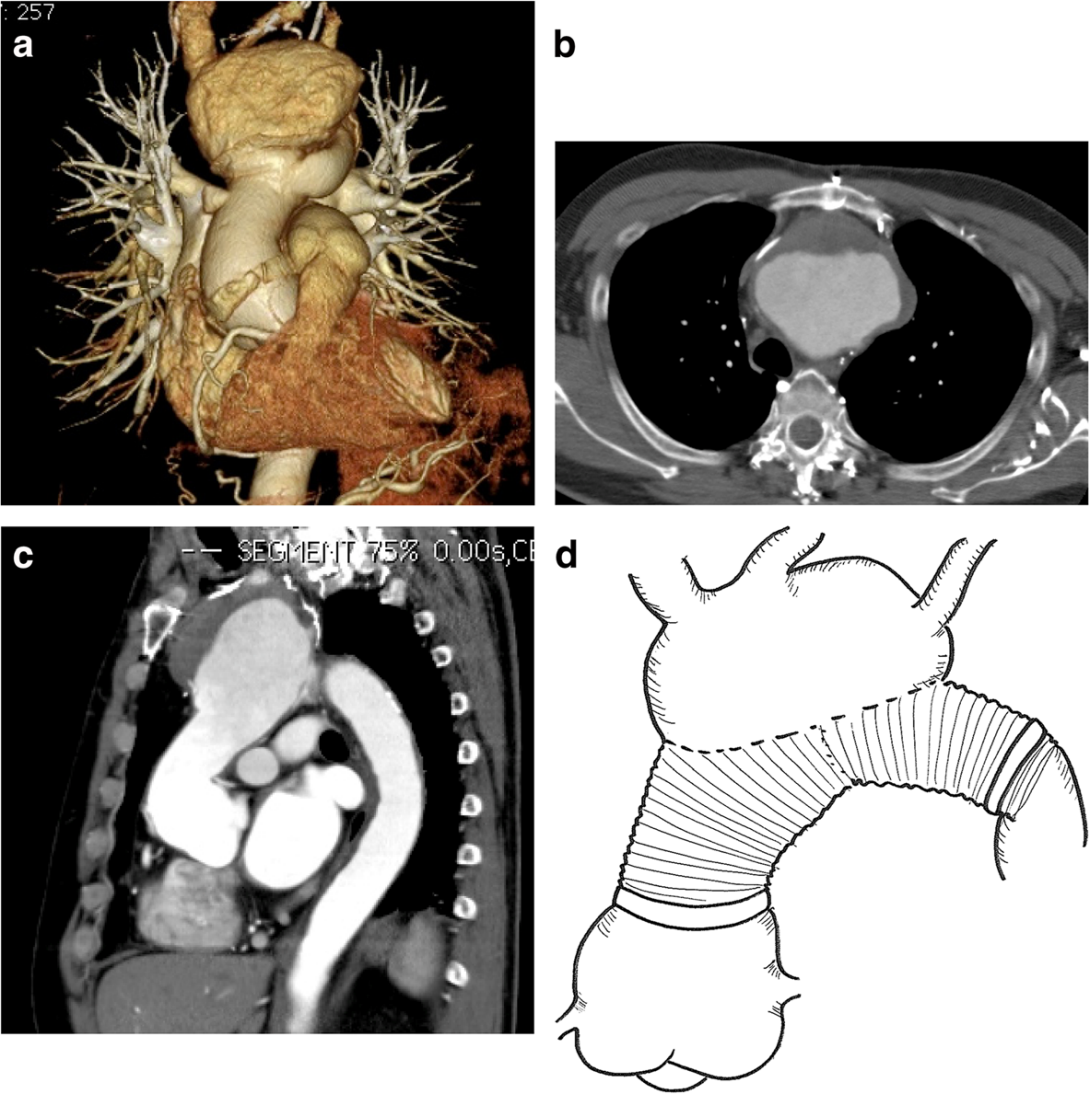
Multidetector computed tomography (MDCT) 11 years after the previous surgery. The MDCT demonstrates a giant arch anastomotic pseudoaneurysm, aortic root enlargement and coronary artery stenosis, and shows no re-dissection in the pseudoaneurysm. **a** Synthetic image, (**b**-**c**) axial view, and (**d**) scheme of the aneurysm

Contrast-enhanced CT **(**Fig. 3**)** showed that a giant arch pseudoaneurysm (81 mm wide) had formed in the island-shaped arch branch. No false lumen was observed in the aneurysm, and its enlargement was omnidirectional. The diameter of the aortic root was also enlarged to approximately 56 mm in size. In addition, coronary angiography indicated that there was 99% stenosis of the left anterior descending coronary artery at #7, collateral circulation from the right coronary artery, and 75% stenosis of the left circumflex artery at #13.

Based upon these observations, the patient underwent a repeat total arch replacement, aortic root replacement (i.e., Bentall procedure), and coronary artery bypass grafting [the left internal thoracic artery (LITA) and saphenous vein graft (SVG) were used]. Under general anesthesia, extracorporeal perfusion was initiated. We re-performed a median sternotomy and successfully opened the thorax without causing any damage to the aneurysm, while gradually decreasing the rectal temperature to 28 °C while sending blood to the right femoral artery and right axillary artery, while removing blood from the right femoral vein. We were able to switch the cannula from the femoral vein to the superior and inferior vena cava, as needed. After removing the previously used vascular graft, the coronary ostia on the right and left were confirmed and excised in the shape of a button. A 23 mm Carpentier-Edwards Perimount (CEP)® bovine pericardial bioprosthesis (Edwards Life Science, Irvine, CA) and a 28 mm Vascutek® Gelweave Valsalva Graft (Terumo Vascutek, Renfrewshire, Scotland, UK) were combined with a running suture, and the skirt portion and the aortic valve annulus were attached with a 3–0 PROLENE running suture (Ethicon, Somerville, NJ, USA), with a felt strip between them. The right and left coronary arteries were continuously sutured with 5–0 PROLENE (Ethicon, Somerville, NJ, USA) for reconstruction of the Carrel patch. When the rectal temperature reached 28 °C, circulation of the lower body was arrested and antegrade selective cerebral perfusion was initiated by the insertion of balloon-occludable catheters to each cervical branch of the aorta. Upon incision of the aneurysm, it was revealed that the inner wall was covered by atheroma, although no false lumen was observed inside the aneurysm or arch branches. After removing as much of the aneurysm as possible, three separate perfusions of cervical branches were performed. On the distal side, another vascular graft was retained and connected to a 26 mm, four-branched synthetic vascular graft (J-graft SHIELD NEO®, Japan Lifeline, Japan) with 3–0 PROLENE (Ethicon, Somerville, NJ, USA) using the open distal technique. After a restart of blood flow from the synthetic vascular graft branch, the left subclavian artery was reconstructed. In addition, the SVG and LITA were sutured to #14 and #8, respectively, and the Valsalva graft and the 26 mm J-graft were attached with running sutures of 4–0 PROLENE (Ethicon, Somerville, NJ, USA). The central side of the SVG was then anastomosed to the J-graft to allow the release of the aorta clamp. Finally, the left common carotid artery and brachiocephalic artery were reconstructed.

The operation was completed under stable hemodynamics. The operation time was 660 min, extracorporeal circulation time was 363 min, aortic clamp time was 172 min, circulatory arrest time was 38 min, the lowest rectal temperature was 27.6 °C, and intraoperative bleeding was 3240 mL.

**Fig. 4:**
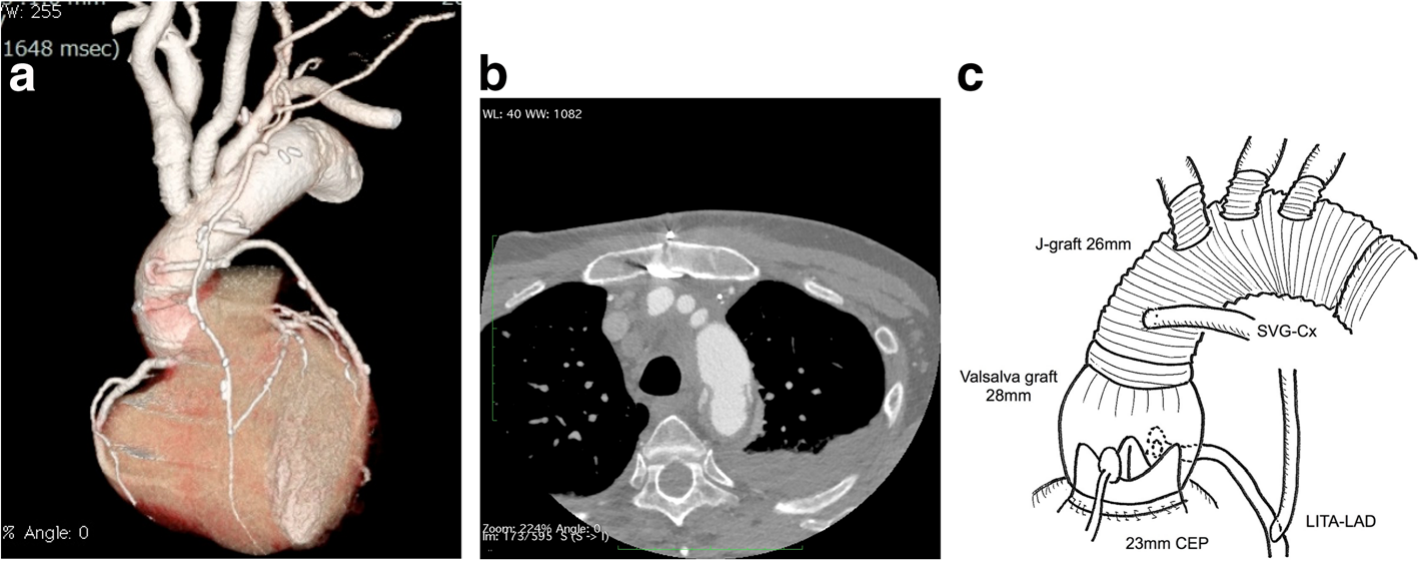
Postoperative multidetector computed tomography (MDCT) scan showing redo total arch replacement, aortic root replacement and coronary artery bypass (LITA-LAD, Ao-SVG-PL). LITA: left internal thoracic artery, LAD: left descending artery, Ao: Aorta, SVG: saphenous vein graft, PL: posterior lateral. **a** Synthetic image, (**b**) axial view, and (**c**) scheme of the aneurysm

Although the patient was re-intubated on the fourth postoperative day after extubation for ventilator failure, he was extubated again and discharged from the ICU on the eighth postoperative day. An indwelling pacemaker was inserted on the 40 s postoperative day due to sick sinus syndrome and the patient was ultimately discharged from the hospital on the fiftieth postoperative day **(**Fig. 4**)**. He has been visiting the hospital as an outpatient and is in favorable condition.

## Discussion

A wide range of arch replacement reconstruction techniques have been reported, including the open distal technique, arch-first technique (AFT), and hypothermic circulatory arrest (HCA)/simple clamping. Moreover, there are several techniques for cerebral perfusion, such as antegrade selective cerebral perfusion (SCP), and retrograde cerebral perfusion (RCP). Therefore, the choice of reconstruction techniques or supplemental devices for perfusion depends on the patient condition, surgeon preference, and institution policy. To reduce the duration of non-physiologic circulation when performing RCP, AFT is employed by some surgeons [6]. AFT shortens cerebral ischemia time and ensures a favorable surgical field. It is also reported to reduce the occurrence of cerebral complications [7]. Other measures have been taken to shorten the cerebral ischemia time, among which arch branched island reconstruction is believed to be a simpler procedure, entailing the requirements of relatively less effort with fewer movements and opportunities for error during operation [8]. In this case, this reconstruction is performed as part of the T-graft technique. In addition to ensuring secure cerebral perfusion, the use of two synthetic vascular grafts with different diameters can provide flexibility in selecting the desired graft diameter when an apparent discrepancy is observed in the proximal and distal sides [9].

Notably, we observed in this case that residual vessel diseases induced the formation of a giant pseudoaneurysm in the eleventh postoperative year. While the formation of a pseudoaneurysm after thoracic aorta synthetic vascular graft replacement is rare, it is a possibly fatal complication once it develops. Reported causes of pseudoaneurysm formation include synthetic vascular graft infection, deterioration/enlargement of synthetic vascular graft, deterioration of suture thread, hypertension, aortic dissection, mechanical stress at the anastomotic site, and tissue necrosis due to the excessive use of gelatin resorcin formalin (GRF) glue [3, 4, 10]. Consequently, the pseudoaneurysm in this case had no obvious cause. Regarding aortic aneurysms, there are some reports that residual weak vessel diseases often necessitate reoperation, even in cases of true aneurysms; thus, a dissected aorta is used in most instances of cervical anastomosis (island reconstruction), including in this case [5]. In the vascular structure reconstructed during the first surgery, blood pumped from the heart was applying pressure directly to the weak anastomotic site, and hence it may have been more likely to form an aneurysm. In cases such as ours, with dissection advanced to the distal aortic arch, employing a reconstruction technique that can remove the weakened aortic wall as much as possible during the first surgery is important.

Treatment using stent grafts for pseudoaneurysms has been reported, but treatment of postoperative aortic arch pseudoaneurysms is difficult due to the complicated shape [11]. However, branch TEVAR is challenging in terms of device preparation. In the debranching and TEVAR technique, re-thoracotomy is necessary, and difficulties in placement and anastomosis of the debranched graft, plus in ensuring exposure of the carotid artery, are to be expected in the case of a giant pseudoaneurysm. In this case, repair with re-thoracotomy was performed since enlargement of the aortic root and complications of other coronary diseases were observed in addition to the presence of non-anatomical reconstruction using the T-graft technique.

During reoperation for thoracic aortic aneurysm, the patient will be at risk if the pseudoaneurysm is ruptured during a median sternotomy; hence, the re-operation is more carefully performed than a general thoracotomy. The effectiveness of extracorporeal circulation using the femoral artery and vein with HCA in performing reoperation for thoracic aortic aneurysm has been reported; thus, it should always be a possibility for consideration, although operative invasiveness must be taken into account [10, 12, 13].

Although five- to 10-year operative outcomes of thoracic aortic surgeries have improved over time, they are still poorly understood due to the scarcity of reports of long-term outcomes over 10 years. In the increasingly aging population, promising long-term prognoses are anticipated, even in patients with Marfan syndrome or those with aortic disease that developed at a relatively young age. As such, reconstruction techniques must be examined in consideration of longer prognoses in the future.

## Conclusions

We experienced a case requiring reoperation for aortic arch pseudoaneurysm 11 years after a total arch replacement with island reconstruction for chronic aortic dissection. We believe that sufficient examination of reconstruction techniques is necessary, particularly in cases of relatively young patients who are expected to have a favorable long-term prognosis.

## Abbreviations

AFT: Arch-first technique
GRF: Gelatin resorcin formalin
HCA: Hypothermic circulatory arrest
LITA: Left internal thoracic artery
RCP: Retrograde cerebral perfusion
SCP: Selective cerebral perfusion
SVG: Saphenous vein graft
TEVAR: Thoracic endovascular aortic repair
